# Mild hyponatremia is associated with low skeletal muscle mass, physical function impairment, and depressive mood in the elderly

**DOI:** 10.1186/s12877-020-01955-4

**Published:** 2021-01-06

**Authors:** Chisato Fujisawa, Hiroyuki Umegaki, Taiki Sugimoto, Satoshi Samizo, Chi Hsien Huang, Haruki Fujisawa, Yoshihisa Sugimura, Masafumi Kuzuya, Kenji Toba, Takashi Sakurai

**Affiliations:** 1 Departments of Community Healthcare and Geriatrics, Graduate School of Medicine, Nagoya University, 65 Tsurumai-cho, Showa-ku, Nagoya City, Aichi 466-8550 Japan; 2 Center for Comprehensive Care and Research on Memory Disorders, National Center for Geriatrics and Gerontology, Obu, Japan; 3 Department of Palliative Medicine, Seirei Hospital, Nagoya, Japan; 4 Department of Endocrinology and Metabolism, Fujita Health University, Toyoake, Japan

**Keywords:** Balance impairment, Electrolyte disorder, Grip strength, Depression, Gait instability, Hyponatremia

## Abstract

**Background:**

Mild hyponatremia (serum sodium 130–135 mEq/L) is a common electrolyte disorder in the elderly. However, its association with both sarcopenia and cognitive function remains to be clarified. Therefore, here we investigated the association of mild hyponatremia with skeletal muscle mass, physical function, and cognitive function in the elderly.

**Methods:**

We enrolled 75 participants with mild hyponatremia and 2907 with normonatremia (serum sodium, 136–145 mEq/L) aged ≥70 years who visited the Memory Disorder Outpatient Center of Japan’s National Center for Geriatrics and Gerontology. Skeletal muscle mass index (SMI), grip strength (GS), walking speed (WS), one-leg standing (OLS) test times, and neuropsychological test scores were determined.

**Results:**

One-way analysis of covariance showed that elderly participants with mild hyponatremia had lower SMI (7.1 ± 0.2, 7.2 ± 0.2 kg/m^2^, *p* = 0.04), weaker GS (19.1 ± 1.9 vs 21.4 ± 1.8 kg, *p* = 0.01), slower WS (0.9 ± 0.1 vs 1.1 ± 0.1 m/s, *p* = 0.001), and higher GDS- 15 score (6.4 ± 0.9 vs 5.2 ± 0.9, *p* = 0.002) than those with normonatremia. Multiple logistic regression analysis indicated that mild hyponatremia was independently associated with sarcopenia (odds ratio [OR]: 2.2, *p* = 0.02), slower WS (OR: 5.3, *p* = 0.04) and shorter OLS time (OR: 2.5, *p* = 0.02) as well as with severe depressive mood (OR: 2.6 *p* = 0.006) but not with SMI (OR: 1.6, *p* = 0.2) or GS (OR: 1.9, *p* = 0.09).

**Conclusions:**

Our results suggest that elderly people with even mild hyponatremia had physical function impairment and depressive mood.

## Background

Hyponatremia is the most common electrolyte disorder in the elderly. It occurs in about 7% of all otherwise healthy people aged 55 years or more, with incidence increasing significantly with age. Hyponatremia in the elderly has many causes, including use of medication such as diuretics and antidepressants, syndrome of inappropriate antidiuretic hormone secretion, malnutrition, and comorbidities such as cardiac disease [1]. In most cases though, the symptoms of hyponatremia are mild and related symptoms are subtle, so it often goes unnoticed by patients and physicians.

However, recent studies suggest that asymptomatic hyponatremia is associated with poor prognosis in the elderly. Largely asymptomatic mild-to-moderate hyponatremia was reported to be associated with gait instability, falls, and fractures [2–4]. Also, even after considering the many etiologies of hyponatremia that may contribute to poor prognosis among the elderly, hyponatremia itself is considered to be an independent risk factor for poor prognosis [2]. Attention deficit has also been reported in both humans and rats with mild-to-moderate hyponatremia [3, 5]. Thus, it seems plausible that asymptomatic hyponatremia contributes to poor prognosis through, for example, falls or fractures probably as a result of impaired attention, posture, and gait.

To date, most studies on the association between physical function and hyponatremia have involved patients with moderate hyponatremia (serum sodium [SNa] < 130 mEq/L) [3]. So, it remains unclear whether there is an association between mild hyponatremia (SNa 130–135 mEq/L), which constitutes most cases of hyponatremia, and both physical and cognitive function in the elderly. In clinical practice, physicians are often hesitant to treat asymptomatic mild hyponatremia with SNa ≥ 130 mEq/L. However, increased mortality has been reported in patients hospitalized with even mild hyponatremia (SNa 130–134 mEq/L) [6]. Thus, we hypothesized that elderly people with even mild hyponatremia, not just those with mild-to-moderate hyponatremia, have physical or cognitive dysfunction such as impaired balance or executive dysfunction. We also hypothesized that there is an association between mild hyponatremia and lower skeletal muscle mass (SMM) in this population based on a report that found an association between chronic hyponatremia and decreased SMM in rats [7].

Because homeostatic responses to environmental challenges decline with aging, hyponatremia is frequently diagnosed in the elderly. We anticipate that more frail elderly patients will present with mild hyponatremia into the future as society ages. Thus, if elderly people with mild hyponatremia tend to have physical or cognitive dysfunction, then we should consider ways to prevent and manage this condition. In this study, we investigated the association of mild hyponatremia with SMM, physical function, and neuropsychological test scores in the elderly.

## Methods

### Participants

We enrolled 2962 elderly participants with normonatremia (SNa 136–145 mEq/) and 75 with hyponatremia (SNa 130–135 mEq/L) aged ≥70 years who visited the National Center for Geriatrics and Gerontology (NCGG) for assessment of memory disorder between September 2010 and November 2017. None of the participants had any severe symptoms such as nausea, vomiting, confusion, or seizures, and all were able to carry out at least 1 of the following 3 physical function tests: grip strength (GS), walking speed (WS), and the one-leg standing (OLS) test. To eliminate pseudohyponatremia and the effect of disease on physical or cognitive function, the following inclusion criteria were adopted: (1) no history of stroke; (2) no pseudohyponatremia (TP ≤ 8.0, Glu ≤ 400, TH < 500, and T-cho ≤ 280); and (3) not taking medication for Parkinson’s disease (e.g., L-dopa, dopamine agonist, trihexyphenidyl, and amantadine).

The study was approved by the NCGG Ethics Committee. All participants provided informed consent before enrollment in the study.

### Clinical measures

The following self-reported and caregiver-reported measures were obtained based on previous studies [3, 4, 6, 8, 9]: age, sex, and years of education, alcohol consumption, body mass index (BMI), history of cardiac disease, diabetes, liver disease, lung disease, depression, chronic kidney disease (CKD), cancer, dementia diagnosis, joint pain, visual impairment, hearing impairment, diuretic use, antibiotics use (e.g., clarithromycin, levofloxacin, and minocycline hydrochloride), proton pump inhibitor use, non-steroidal anti-inflammatory drug (NSAID) or acetaminophen use, antiepileptic use (e.g., sodium valproate, and carbamazepine), antipsychotic use (e.g., risperidone, haloperidol, and aripiprazole), noradrenergic and specific serotonergic antidepressant (NaSSA) use, selective serotonin reuptake inhibitor (SSRI) or serotonin noradrenaline reuptake inhibitor (SNRI) use, benzodiazepine use (e.g., alprazolam, brotizolam, etizolam, and flunitrazepam), and frequency of physical activity. Those who consumed > 60.0 g/day of alcohol were defined as heavy drinkers. No exercise per week was defined as inactivity. Potassium, calcium, brain natriuretic peptide, estimated glomerular filtration rate, C-reactive protein (CRP), hemoglobin (Hb), thyroid-stimulating hormone, and vitamin B12 were checked from their blood samples. Anemia was defined their serum Hb was < 11.0 g/dL. Hypothyroidism was defined as a serum thyroid-stimulating hormone level of 10 μIU/mL or less. Vitamin B12 deficiency was defined as a serum vitamin B12 level of < 200 pg/mL.

### Assessment of SMM, physical function, and sarcopenia

Body weight and SMM were assessed using a multi-frequency body composition analyzer (MC-180 Multi-Frequency Body Composition Analyzer; Tanita, Tokyo, Japan), which is a scale that uses bioelectrical impedance analysis to determine body composition. We calculated BMI (body weight [kg]/height squared [m^2^]) and SMM index (SMI; i.e., SMM [kg]/height squared [m^2^]). Low SMM was defined as SMI < 7.0 kg/m^2^ in men and < 5.7 kg/m^2^ in women [10].

Physical function was assessed by measuring GS, WS, and OLS time. Muscle strength was from upper extremity strength measured with a digital force gauge (ZP 500 N; Imada, Toyohashi, Japan) [11]. The best GS (kg) was determined, with low muscle strength defined as < 28 kg in men and < 18 kg in women, in accordance with the Asian Working Group for Sarcopenia (AWGS) criteria [10]. Velocity was determined by measuring normal WS, with poor physical performance defined as WS < 1.0 m/s [10]. Postural function was assessed using the OLS test, which measures the time in seconds (up to a maximum of 60 s) that participants can stand unassisted on one leg for as long as possible with their eyes open. The best score was used for the analysis [8]. Impaired postural function was defined as OLS time < 15 s according to the locomotive syndrome criteria [12]. Sarcopenia was defined as low SMM, low muscle strength, and/or low physical function [10].

### Assessment of cognitive function

Cognitive function was assessed with the Mini-Mental State Examination (MMSE) [13], executive function with the Frontal Assessment Battery (FAB) [14], working memory with the Digit Span subtest (forward and backward) of the Wechsler Adult Intelligence Scale [15], attention with the category fluency subtest of the Hasegawa Dementia Rating Scale-Revised [16], and memory with the Logical Memory II subtest of the Wechsler Memory Scale-Revised (WMS-R) [17].

Cognitive impairment was indicated by MMSE score ≤ 23, FAB score ≤ 11 [18], Digit Span forward score ≤ 5, Digit Span backward score ≤ 3, category fluency test score ≤ 5, and WMS-R Logical Memory II subtest raw score ≤ 8 for > 16 years of education, ≤ 4 for 8–15 years of education, and ≤ 2 for 0–7 years of education [19]. Depressive mood was assessed using the Geriatric Depression Scale 15 (GDS-15) [20], with a score ≥ 10 indicating severe depressive mood [21]. All of the cognitive test results were reviewed by neuropsychologists.

### Statistical analysis

The Chi-squared test and Mann–Whitney’s U test were used to investigate differences in characteristics between mild hyponatremia and normonatremia. One-way analysis of covariance (ANCOVA) was used to identify significant differences between the 2 groups in SMI, the 3 physical function test scores, and all the neuropsychological test scores after controlling for covariates. Then, multiple logistic regression analysis was conducted to examine the correlation between mild hyponatremia and sarcopenia, SMI, physical function test scores, and all the neuropsychological test scores. Cutoff scores for these dependent variables were based on the studies mentioned above. All the variables whose *p*-value was < 0.2 in Table 1 were selected as covariates for both ANCOVA and multiple regression analysis as shown under Tables 3 and 4. All analyses were performed using SPSS Statistics for Windows ver. 26.0 (IBM Corp., Armonk, NY). A *p*-value < 0.05 was considered significant.

**Table 1:** Participant characteristics

	n	Mild hyponatremia group	n	Normonatremia group	***p***
Age, years	75	82.0 (76.0–84.0)	2907	79.0 (75.0–83.0)	0.03
Female, n (%)	75	36 (48.0)	2907	1833 (63.1)	0.008
Education, years	75	9.0 (9.0–12.0)	2869	9.0 (9.0–12.0)	0.8
Heavy drinker^a^, n (%)	74	1 (1.4)	2879	55 (1.9)	0.7
Body mass index, kg/m^2^	75	21.0 (19.1–23.6)	2905	21.8 (19.8–24.0)	0.09
History of cardiac disease, n (%)	75	15 (20.0)	2907	365 (12.6)	0.06
History of diabetes, n (%)	75	36 (48.0)	2907	1049 (36.1)	0.03
History of liver disease, n (%)	75	1 (1.3)	2907	61 (2.1)	0.6
History of lung disease, n (%)	75	4 (5.3)	2907	119 (4.1)	0.6
History of depression, n (%)	75	2 (2.7)	2907	106 (3.6)	0.7
History of CKD, n (%)	75	33 (44.0)	2905	1006 (34.6)	0.1
History of cancer, n (%)	75	9 (12.0)	2907	240 (8.3)	0.2
Dementia, n (%)	75	45 (60.0)	2907	1516 (52.1)	0.2
Joint pain, n (%)	74	31 (41.9)	2890	1173 (40.6)	0.8
Visual impairment, n (%)	74	37 (50.0)	2888	1607 (55.6)	0.4
Hearing impairment, n (%)	74	47 (63.5)	2883	1524 (52.9)	0.07
Diuretics use, n (%)	75	6 (8.0)	2907	129 (4.4)	0.1
Antibiotics, n (%)	75	0 (0.0)	2907	34 (1.2)	0.3
Proton pump inhibitor use, n (%)	75	6 (8.0)	2907	208 (7.2)	0.8
NSAID or acetaminophen use, n (%)	75	4 (5.3)	2907	127 (4.4)	0.7
Antiepileptic use, n (%)	75	2 (2.7)	2907	11 (0.4)	0.003
Antipsychotic use, n (%)	75	0 (0.0)	2907	17 (58.5)	0.5
NaSSA use, n (%)	75	1 (1.3)	2907	6 (0.2)	0.05
SSRI or SNRI use, n (%)	75	5 (6.7)	2907	51 (1.8)	0.002
Benzodiazepine use, n (%)	75	12 (16.0)	2907	254 (8.7)	0.03
Inactivity, n (%)	74	21 (28.4)	2864	957 (33.4)	0.4
Potassium, mEq/L	75	4.4 (4.1–4.6)	2907	4.2 (2.9–4.4)	< 0.001
Calcium, mg/dL	75	9.4 (9.1–9.7)	2901	9.5 (9.2–9.8)	0.4
BNP, pg/mL	38	30.1 (16.5–64.9)	1562	31.8 (16.3–60.2)	0.9
eGFR, mL/min/1.73m^2^	75	62.1 (52.2–80.9)	2905	63.3 (54.0–73.2)	0.6
CRP, mg/dL	74	0.07 (0.04–0.2)	2881	0.05 (0.03–0.1)	0.02
Anemia^b^, n (%)	75	10 (13.3)	2907	195 (6.7)	0.03
Hypothyroidism^c^, n (%)	75	2 (2.7)	2898	10 (0.3)	0.002
Vitamin B12 deficiency^d^, n (%)	63	1 (1.6)	2535	59 (2.3)	0.7

Values are median (interquartile range) or number (percentage). Differences were assessed using the Kruskal–Wallis test for continuous variables or the Chi-squared test for categorical variables

*Abbreviations*: *BNP* Brain natriuretic peptide, *CRP* C-reactive protein, *eGFR* Estimated glomerular filtration rate, *NSAID* Non-steroidal anti-inflammatory drug, *NaSSA* Noradrenergic and specific serotonergic antidepressant, *SNRI* Serotonin noradrenaline reuptake inhibitor, *SSRI* Selective serotonin reuptake inhibitors. ^a^ > 60 g/day of alcohol, ^b^Hemoglobin < 11.0 g/dL, ^c^thyroid-stimulating hormone ≤10μIU/mL, ^d^vitamin B12 < 200 pg/mL

## Results

During the study period, 7580 individuals aged ≥70 years visited the NCGG Memory Disorder Outpatient Center. Of them, 3097 met the inclusion criteria: 8 had moderate or more severe hyponatremia (SNa < 130 mEq/L), 75 had mild hyponatremia (SNa 130–135 mEq/L), 2907 had normonatremia (SNa 136–145 mEq/L), and 107 had hypernatremia (SNa > 145 mEq/L). Thus, 75 participants with mild hyponatremia and 2907 with normonatremia were included in this study.

The characteristics of hyponatremia and normonatremia are summarized for all participants in Table 1. The mild hyponatremia group had significantly older age (median [interquartile range], 82.0 [76.0–84.0] vs 79.0 [75.0–83.0] years), fewer women (48.0% vs 63.1%), higher prevalence of history of diabetes (48.0% vs 36.1%), higher antiepileptic use (2.7% vs 0.4%), higher SSRI or SNRI use (6.7% vs 1.8%), higher benzodiazepine use (16.0% vs 8.7%), higher potassium (4.4 [4.1–4.6] vs 4.2 [2.9–4.4] mEq/L), higher CRP (0.07 [0.04–0.2] vs 0.05 [0.03–0.1] mg/dL), and higher frequency of anemia (13.3% vs 6.7%) and hypothyroidism (2.7% vs 0.3%) compared with the normonatremia group. As shown in Table 2, the mild hyponatremia group also had more cases of sarcopenia, weaker GS, slower WS, shorter OLS time, and higher GDS-15 score.

**Table 2:** Difference in physical and cognitive function between the groups

	n	Mild hyponatremia group	n	Normonatremia group	***p***
Sarcopenia, n (%)	52	34 (65.4)	2110	928 (44.0)	0.002
SMI, kg/m^2^	51	6.4 (5.7–6.9)	2418	6.4 (5.7–7.2)	0.5
Physical function tests					
Grip strength, kg	48	17.7 (12.9–22.2)	1972	19.7 (15.2–25.0)	0.04
Walking speed, m/s	17	0.7 (0.5–0.9)	704	1.0 (0.8–1.2)	< 0.001
One-leg standing time, s	54	5.0 (2.6–13.1)	2546	8.6 (3.7–21.9)	0.008
Neuropsychological tests					
MMSE	75	20.0 (17.0–25.0)	2901	21.0 (17.0–25.0)	0.4
FAB	34	9.0 (7.0–12.0)	1732	10.0 (8.0–12.0)	0.6
Digit Span forward	38	5.0 (5.0–6.0)	1775	5.0 (4.8–6.0)	0.9
Digit Span backward	38	3.0 (3.0–4.0)	1761	3.0 (3.0–4.0)	0.8
Category fluency	75	8.0 (6.0–10.0)	2899	8.0 (5.0–10.0)	0.3
Logical memory II	37	3.0 (3.0–12.0)	1780	3.0 (3.0–11.0)	0.3
GDS-15	72	4.0 (2.0–6.0)	2893	5.0 (3.0–8.0)	0.002

Values are median (interquartile range) or number (percentage). Differences were assessed using the Kruskal–Wallis test for continuous variables or the Chi-squared test for categorical variables

*Abbreviations*: *FAB* Frontal Assessment Battery, *GDS-15* 15-item Geriatric Depression Scale, *MMSE* Mini-Mental state examination, *SMI* Skeletal muscle mass index

ANCOVA showed that mild hyponatremia had a significant effect on SMI (*F* [1, 2393) = 4.2; *p* = 0.04), GS (*F* [1, 1951] = 6.6; *p* = 0.01), WS (*F* [1, 672] = 11.9; *p* = 0.001), and GDS-15 score (*F* [1, 2883] = 9.7; *p* = 0.002) after controlling for covariates, and a nearly significant effect on OLS time (*F* [1, 2527] = 3.6; *p* = 0.06) (Table 3).

**Table 3:** ANCOVA results for the comparison of skeletal mass index, physical function, and psychological tests between the groups

		Mild hyponatremia group		Normonatremia group			
	n	Adjusted mean ± SD	n	Adjusted mean ± SD	F	***p***	η^**2**^
Skeletal muscle mass index, kg/m^2^	50	7.1 ± 0.2	2361	7.2 ± 0.2	4.2	0.04	0.002
Grip strength, kg	46	19.1 ± 1.9	1923	21.4 ± 1.8	6.6	0.01	0.003
Walking speed, m/s	16	0.9 ± 0.1	673	1.1 ± 0.1	11.9	0.001	0.02
One-leg standing test, s	53	7.5 ± 4.9	2492	11.8 ± 4.6	3.6	0.06	0.001
Mini–Mental State Examination	73	20.3 ± 1.5	2839	20.5 ± 1.4	0.06	0.8	< 0.001
Frontal Assessment Battery	33	7.5 ± 1.2	1689	7.5 ± 1.1	0.001	0.99	< 0.001
Digit Span forward	37	4.7 ± 0.4	1732	4.8 ± 0.4	0.4	0.5	< 0.001
Digit Span backward	37	2.2 ± 0.4	1718	2.2 ± 0.4	0.002	0.96	< 0.001
Verbal fluency	73	7.7 ± 0.6	2838	7.5 ± 0.6	0.003	0.95	< 0.001
Logical memory II	36	4.8 ± 5.7	1737	3.7 ± 5.2	0.2	0.7	< 0.001
15-item Geriatric Depression Scale	71	6.4 ± 0.9	2830	5.2 ± 0.9	9.7	0.002	0.003

η^2^, the effect size

Covariates adjusted for were age, sex, body mass index, history of heart disease, diabetes, CKD, hearing impairment, diuretic use, antiepileptic use, NaSSA use, SSRI or SNRI use, benzodiazepine use, serum potassium, C-reactive protein, anemia, and hypothyroidism

*Abbreviations*: *SD* Standard deviation

The results of multiple logistic regression using each cutoff score are shown in Table 4. After entering all variables written under Table 4 into multivariable models 2, the elderly with mild hyponatremia were significantly more likely to have sarcopenia (odds ratio [OR], 2.2; 95% confidence interval [CI], 1.1–4.4; *p* = 0.02), poor physical performance as indicated by slower WS (OR, 5.3; 95% CI, 1.1–25.4; *p* = 0.04), impaired postural balance (OR, 2.5; 95% CI, 1.2–5.5; *p* = 0.02), and severe depressive mood (OR, 2.6; 95% CI, 1.3–5.2; *p* = 0.006) and tended to have low muscle mass as indicated by weaker GS (OR, 1.9; 95% CI; 0.9–3.8; *p* = 0.09). However, mild hyponatremia was not significantly associated with lower SMI (OR, 1.6; 95% CI, 0.8–3.2; *p* = 0.2). There was no overfitting, and goodness of fit (Hosmer-Lemeshow test) indicated that each logistic model fit well.

**Table 4:** Results of logistic regression analysis for predicting sarcopenia, skeletal muscle mass, and physical and cognitive functions

Dependent variables	Model 1			Model 2		
	n	OR (95% CI)	***p***	n	OR (95% CI)	***p***
Sarcopenia	2244	2.2 (1.2–4.0)	0.008	2107	2.2 (1.1–4.4)	0.02
Low skeletal muscle mass index^a^	2561	1.7 (1.0–3.0)	0.07	2411	1.6 (0.8–3.2)	0.2
Low muscle strength^b^	2097	2.2 (1.1–4.4)	0.03	1968	1.9 (0.9–3.8)	0.09
Poor physical performance; Walking speed < 1 m/s	747	3.5 (1.0–13.0)	0.06	689	5.3 (1.1–25.4)	0.04
One-leg standing test < 15 s	2695	2.3 (1.1–4.8)	0.03	2541	2.5 (1.2–5.5)	0.02
Cognitive disturbance; MMSE < 24	3091	1.1 (0.7–1.9)	0.6	2912	1.1 (0.7–1.9)	0.7
Executive dysfunction; FAB < 12	1831	1.0 (0.5–2.3)	0.95	1722	0.9 (0.4–2.1)	0.8
Poor walking memory; Digit Span forward < 6	1883	0.9 (0.5–1.9)	0.8	1769	0.9 (0.4–1.9)	0.8
Poor walking memory; Digit Span backward < 4	1869	1.1 (0.5–2.1)	0.8	1755	1.0 (0.5–2.1)	0.96
Poor attention; the category fluency subtest of the HDS-R < 6	3088	1.1 (0.6–1.8)	0.8	2911	1.0 (0.6–1.7)	0.99
Memory disorder^c^	1889	0.7 (0.3–1.5)	0.4	1772	0.8 (0.4–1.9)	0.7
Severe depression; GDS-15 ≥ 10	3078	2.7 (1.4–5.2)	0.004	2901	2.6 (1.3–5.2)	0.006

Model 1. Adjusted for age and sex

Model 2. In the binary logistic regression analysis, all variables were entered into the multivariable model (age, sex, body mass index, history of heart disease, diabetes, CKD, hearing impairment, diuretic use, antiepileptic use, NaSSA use, SSRI or SNRI use, benzodiazepine use, serum potassium, C-reactive protein, anemia, and hypothyroidism)

*Abbreviations*: CI Confidence interval, *CKD* Chronic kidney disease, *FAB* Frontal Assessment Battery, *GDS-15* 15-item Geriatric Depression Scale, *HDS-R* Hasegawa Dementia Rating Scale-Revised, *MMSE* Mini-Mental State Examination, *NaSSA* Noradrenergic and specific serotonergic antidepressant, *OR* Odds ratio, *SNRI* Serotonin noradrenaline reuptake inhibitor, *SSRI* Selective serotonin reuptake inhibitors, *WMS-R* Wechsler Memory Scale-Revised

^a^Low skeletal muscle mass was defined as skeletal muscle mass index < 7.0 kg/m^2^ in men and < 5.7 kg/m^2^ in women

^b^Reduced grip strength was defined as < 28 kg for men and < 18 kg for women

^c^Raw WMS-R II score ≤ 8/9 for > 16 years of education, ≤ 4/5 for 8–15 years of education, ≤ 2/3 for 0–7 years of education

## Discussion

### Correlation between mild hyponatremia and SMM and physical function

The mild hyponatremia group had significantly lower SMI, weaker GS, and slower WS, and nearly significantly shorter OLS time compared with the normonatremia group, even after controlling for covariates. When using the AWGS criteria for sarcopenia, the mild hyponatremia group had a significantly higher risk of sarcopenia and worse physical performance and impaired balance, and a marginally significant higher risk of worse muscle strength but was not at risk of having a lower SMI.

The present study demonstrated that elderly people with even mild hyponatremia had gait disturbances and balance impairment. Previous studies demonstrated that chronic hyponatremia causes gait disturbances in both rats [5] and humans [2]. The mechanism behind these observations is yet to be determined. However, for the mechanism whereby hyponatremia contributes to gait dysfunction and balance impairment, if we look at it from the viewpoint of the central nervous system (CNS), the brain cells adapt to hyponatremia by swelling with the loss of osmolytes, such as glutamate [22], which is a neurotransmitter involved in gait and balance function [4]. Rerelease of glutamate from the cytosol to extracellular space contributes to excessive activation of neuronal glutamate receptors, causing excitotoxic cell damage and death in the CNS [23]. However, it remains to be elucidated what severity of hyponatremia, and particularly whether mild hyponatremia, causes these consequences, which could lead to slow WS and balance dysfunction.

On the other hand, we found that muscle strength as measured by GS was significantly different between the mild hyponatremia and normonatremia groups of elderly participants, but when using the AWGS cutoff value for sarcopenia, the logistic regression model showed that the association between mild hyponatremia and GS was not significant. Although Cairns et al. observed a 10% decrease in muscle strength when the sodium concentration was decreased from 147 to 60 mmol/L in mice [24], Vandergheynst et al. did not observe any significant difference in GS when the sodium concentration was increased from 127.7 ± 2.5 mmol/L to 136.1 ± 1.8 mmol/L in humans [25]. Mild hyponatremia itself is correlated with muscle strength, but it’s impact on GS may be insufficient to bring the GS under the cutoff point.

Our study revealed that elderly individuals with even mild hyponatremia had poor physical function. A previous study reported that elderly patients with similar severity of hyponatremia were much more sensitive to alterations in gait tests compared with younger patients [26]. These findings imply that elderly individuals with even mild hyponatremia may be at risk of physical dysfunction.

In clinical practice, it is particularly important to know that the elderly with mild hyponatremia have low physical function because, as mentioned above, mild hyponatremia often goes unnoticed by patients and physicians and physicians are often hesitant to treat asymptomatic mild hyponatremia with SNa ≥130 mEq/L. However, a previous study reported a significant improvement in activities of daily living and cognition among geriatric patients effectively treated for hyponatremia [27]. Mild hyponatremia in the elderly deserves more of our attention. Additional longitudinal studies are needed to strengthen the evidence on the severity of hyponatremia that affects physical function in the elderly and the underlying mechanism.

Although we included many covariates, we might have missed some unmeasured or unknown potential confounders. In addition, we did not determine the causes or chronicity of mild hyponatremia. The duration of hyponatremia or severity of comorbidities may be additional risk factors for physical dysfunction or depressive mood. Hyponatremia could be a marker of severity in these unmeasured underlying illnesses and might signal the presence of physiologic derangements or comorbidities.

Regarding low muscle mass in elderly with mild hyponatremia, hyponatremia tended to lead to progressive SMM loss in rats [7]. Hyponatremia has also been often observed in patients who are malnourished and in the elderly with sarcopenia [28]. In the present study, although there was no significant difference in BMI between the elderly with mild hyponatremia and those with normonatremia, the elderly with mild hyponatremia might have had low protein intake and/or vitamin D deficiency. However, the impact of these factors on muscle strength may be insufficient to bring the SMI score under the cutoff point for mild hyponatremia.

### Association between mild hyponatremia and depressive mood

This study also found that mild hyponatremia was associated with depressive mood, but not with other neuropsychological tests scores. To our knowledge, this study is the first to report an association between mild hyponatremia and depressive mood in the elderly. Previously, the efficacy of tolvaptan was evaluated in patients with hyponatremia. Serum sodium concentrations increased more in the tolvaptan group than in the placebo group and a significant improvement was observed in the tolvaptan group on the Mental Component Summary (for vitality, social functioning, emotionally limited accomplishment, calmness, and sadness) of the Medical Outcomes Study 12-item Short-Form General Health Survey [29]. The mechanism has yet to be determined, but some studies have suggested a correlation between depression and glutamate, whose release from brain cells to the extracellular space may be mediated by hyponatremia [30, 31]. Anti-glutamate agents are known to have antidepressant effects in patients with depression [32]. Alternatively, elderly individuals with depressive mood might not have sufficient appetite to consume an appropriate amount of dietary sodium.

### Study strengths and limitations

This study used detailed neuropsychological test results and is the first to reveal a correlation between mild hyponatremia and depressive mood. A strength of the study is its large sample, which may have minimized many possible confounders, including pseudohyponatremia, many types of medication, and past histories.

However, there are also some limitations. Despite the large sample size, few individuals with hyponatremia undertook the physical function tests, especially for WS. This left us with a small sample of mild hyponatremia, which did not allow us to adapt another mild hyponatremia cutoff, such as 134 mEq/L. Next, as mentioned above, we might have missed some unmeasured or unknown potential confounders; for example, we did not obtain enough data about locomotor system disease or endocrine disease to consider their potential effects. Lastly, this is a cross-sectional study and the specific mechanisms underlying the association of mild hyponatremia with physical function and depressive mood remain to be investigated.

## Conclusions

In this study, elderly participants with mild hyponatremia had impaired lower skeletal muscle mass, physical function, and depressive mood compared with elderly participants with normonatremia. Mild hyponatremia in the elderly deserves more attention than it currently receives. Additional longitudinal studies with a larger number of participants with hyponatremia are needed to strengthen the evidence on the severity of hyponatremia that affects physical and cognitive function in the elderly.

## Data Availability

The data supporting the conclusion of this article may be made available by the corresponding author upon reasonable request.

## Abbreviations

BMI: Body mass index
CI: Confidence interval
CNS: Central nervous system
FAB: Frontal Assessment Battery
GDS-15: 15-item Geriatric Depression Scale
OLS: One-leg standing
OR: Odds ratio
MMSE: Mini-Mental State Examination
NaSSA: Noradrenergic and specific serotonergic antidepressant
NSAID: Non-steroidal anti-inflammatory drug
SMI: Skeletal muscle mass index
SMM: Skeletal muscle mass
SNRI: Serotonin noradrenaline reuptake inhibitor
SSRI: Selective serotonin reuptake inhibitor
WMS-R: Wechsler Memory Scale-Revised
WS: Walking speed
